# Patterns of Herpes Simplex Virus 1 Infection in Neural Progenitor Cells

**DOI:** 10.1128/JVI.00994-20

**Published:** 2020-07-30

**Authors:** Wenxiao Zheng, Alissa M. Klammer, Jennifer N. Naciri, Jason Yeung, Matthew Demers, Jadranka Milosevic, Paul R. Kinchington, David C. Bloom, Vishwajit L. Nimgaonkar, Leonardo D’Aiuto

**Affiliations:** a Department of Psychiatry, Western Psychiatric Institute and Clinic, University of Pittsburgh School of Medicine, Pittsburgh, Pennsylvania, USA; b Third Xiangya Hospital, Xiangya School of Medicine, Central South University, Changsha, China; c University of Pittsburgh, Department of Infectious Diseases and Microbiology, Pitt Graduate School of Public Health, Pittsburgh, Pennsylvania, USA; d Captis Diagnostics, Pittsburgh, Pennsylvania, USA; e Carnegie Mellon University, Pittsburgh, Pennsylvania, USA; f Department of Ophthalmology, University of Pittsburgh School of Medicine, Pittsburgh, Pennsylvania, USA; g Department of Molecular Microbiology and Genetics, University of Pittsburgh, Pittsburgh, Pennsylvania, USA; h Department of Molecular Genetics & Microbiology, University of Florida College of Medicine, Gainesville, Florida, USA; University of California, Irvine

**Keywords:** acyclovir, brivudin, cognition, herpes simplex virus 1 (HSV-1), neural progenitor cells (NPCs), neurospheres

## Abstract

Herpes simplex virus 1 (HSV-1) can induce damage in brain regions that include the hippocampus and associated limbic structures. These neurogenic niches are important because they are associated with memory formation and are highly enriched with neural progenitor cells (NPCs). The susceptibility and fate of HSV-1-infected NPCs are largely unexplored. We differentiated human induced pluripotent stem cells (hiPSCs) into NPCs to generate two-dimensional (2D) and three-dimensional (3D) culture models to examine the interaction of HSV-1 with NPCs. Here, we show that (i) NPCs can be efficiently infected by HSV-1, but infection does not result in cell death of most NPCs, even at high multiplicities of infection (MOIs); (ii) limited HSV-1 replication and gene expression can be detected in the infected NPCs; (iii) a viral silencing mechanism is established in NPCs exposed to the antivirals (E)-5-(2-bromovinyl)-2′-deoxyuridine (5BVdU) and alpha interferon (IFN-α) and when the antivirals are removed, spontaneous reactivation can occur at low frequency; (iv) HSV-1 impairs the ability of NPCs to migrate in a dose-dependent fashion in the presence of 5BVdU plus IFN-α; and (v) 3D cultures of NPCs are less susceptible to HSV-1 infection than 2D cultures. These results suggest that NPC pools could be sites of HSV-1 reactivation in the central nervous system (CNS). Finally, our results highlight the potential value of hiPSC-derived 3D cultures to model HSV-1–NPC interaction.

**IMPORTANCE** This study employed human induced pluripotent stem cells (hiPSCs) to model the interaction of HSV-1 with NPCs, which reside in the neurogenic niches of the CNS and play a fundamental role in adult neurogenesis. Herein, we provide evidence that in NPCs infected at an MOI as low as 0.001, HSV-1 can establish a latent state, suggesting that (i) a variant of classical HSV-1 latency can be established during earlier stages of neuronal differentiation and (ii) neurogenic niches in the brain may constitute additional sites of viral reactivation. Lytic HSV-1 infections impaired NPC migration, which represents a critical step in neurogenesis. A difference in susceptibility to HSV-1 infection between two-dimensional (2D) and three-dimensional (3D) NPC cultures was observed, highlighting the potential value of 3D cultures for modeling host-pathogen interactions. Together, our results are relevant in light of observations relating HSV-1 infection to postencephalitic cognitive dysfunction.

## Abstract (precis)

This study employed human induced pluripotent stem cells (hiPSCs) to model the interaction of HSV-1 with NPCs, which reside in the neurogenic niches of the CNS and play a fundamental role in adult neurogenesis. Herein, we provide evidence that in NPCs infected at an MOI as low as 0.001, HSV-1 can establish a latent state, suggesting that (i) a variant of classical HSV-1 latency can be established during earlier stages of neuronal differentiation and (ii) neurogenic niches in the brain may constitute additional sites of viral reactivation. Lytic HSV-1 infections impaired NPC migration, which represents a critical step in neurogenesis. A difference in susceptibility to HSV-1 infection between two-dimensional (2D) and three-dimensional (3D) NPC cultures was observed, highlighting the potential value of 3D cultures for modeling host-pathogen interactions. Together, our results are relevant in light of observations relating HSV-1 infection to postencephalitic cognitive dysfunction.

## INTRODUCTION

Herpes simplex virus 1 (HSV-1) causes lifelong latent infections, periodic reactivation of which may result in recurrent disease (1). The annual incidence of herpes simplex encephalitis (HSE) worldwide is 2 to 4 per 1,000,000, with rates of neonatal encephalitis rising in the United States (2–4). While such cases can be treated by antiviral acyclovir therapy, which dramatically reduces the mortality to approximately 25%, over 60% of survivors experience severe long-term neurologic deficits. Memory, both anterograde and retrograde, is often impaired, as well as executive function and language ability (5).

Studies of HSV-1 in the brains of animal models have indicated that the virus has a tropism for the lateral ventricles and hippocampus, which correlates well with the human infection (6). The subventricular zone (SVZ) of the lateral ventricles and subgranular zone (SGZ) of the hippocampus are both rich in neural progenitor cells (NPCs), which play a pivotal role in adult neurogenesis. Our previous studies have shown that cultured human NPCs are susceptible to HSV-1 *in vitro* (7, 8).

However, results from animal models of HSV-1 encephalitis show some discrepancy about the ability of the virus to infect NPCs in the SVZ of mice. NPCs can be defined by the expression of several markers that include nestin, Sox1, Pax6, and Sox2 (9). Rotschafer et al. showed that HSV-1 strain 17syn+ colocalized mainly with MAP2^+^ neurons but not with SOX2^+^ NPCs in the SVZ of BALB/c mice infected intranasally (10). In contrast, Chucair-Elliott et al. demonstrated that 24 h after stereotaxic administration of HSV-1 strain McKrae into the lateral ventricles of C57BL/6 mice, the virus colocalized with nestin-positive NPCs. In the same study, the authors showed a drastic reduction (approximately 50%) of NPCs in the subventricular zone in mice following HSV-1 infection of the cornea. Furthermore, adult murine NPCs were susceptible to HSV-1 infection in a dose-dependent fashion, and this infection resulted in an impairment of neuronal differentiation (11). When a rat organotypic hippocampal slice culture model was used, it was shown that HSV-1 predominantly kills granule cells in the hippocampus (12). However, the effect of HSV-1 on NPCs was not investigated (12). Thus, there are differences in reports of HSV-1 effects after infection of the brain. It is not clear if these are related to viral strain, the rodent model used, or the route of inoculation and likely effective inoculum.

In addition, there is uncertainty about the proportion of NPCs that survive acute HSV-1 infection and what effect infection has on function of surviving NPCs. This point is especially significant given the fundamental importance of hippocampal neurogenesis (13). It is possible that the neurological sequelae in HSE survivors reflect, in part, impaired neurogenesis resulting from the effects of HSV-1 on NPCs. While HSV-1 can become latent in many neuronal types and different sensory and autonomic ganglia, it is not known whether HSV-1 can establish a latent infection in NPCs. Such latency or periodic reactivation from latency could lead to the neurological malfunction and memory deficits seen in HSE patients.

To start to address these questions, we modeled HSV-1–NPC interactions following HSV-1 infection of two-dimensional (2D) and three-dimensional (3D) NPC culture models derived from human-induced pluripotent stem cells (hiPSCs). Our data indicate that HSV-1 can infect NPCs with both productive- and persistent-infection outcomes.

## RESULTS

### 3D cultures of NPCs are less susceptible to HSV-1 than 2D cultures.

We and others have previously shown that NPCs, when cultured as 2D monolayers, are susceptible to HSV-1 (7, 8, 14). Considering the accumulating evidence that 3D cultures provide more physiologically relevant systems for modeling host-pathogen interactions (15), we asked whether the introduction of the “third dimension” to the cultures affects the outcome of the NPCs’ infection with HSV-1.

**FIG 1:**
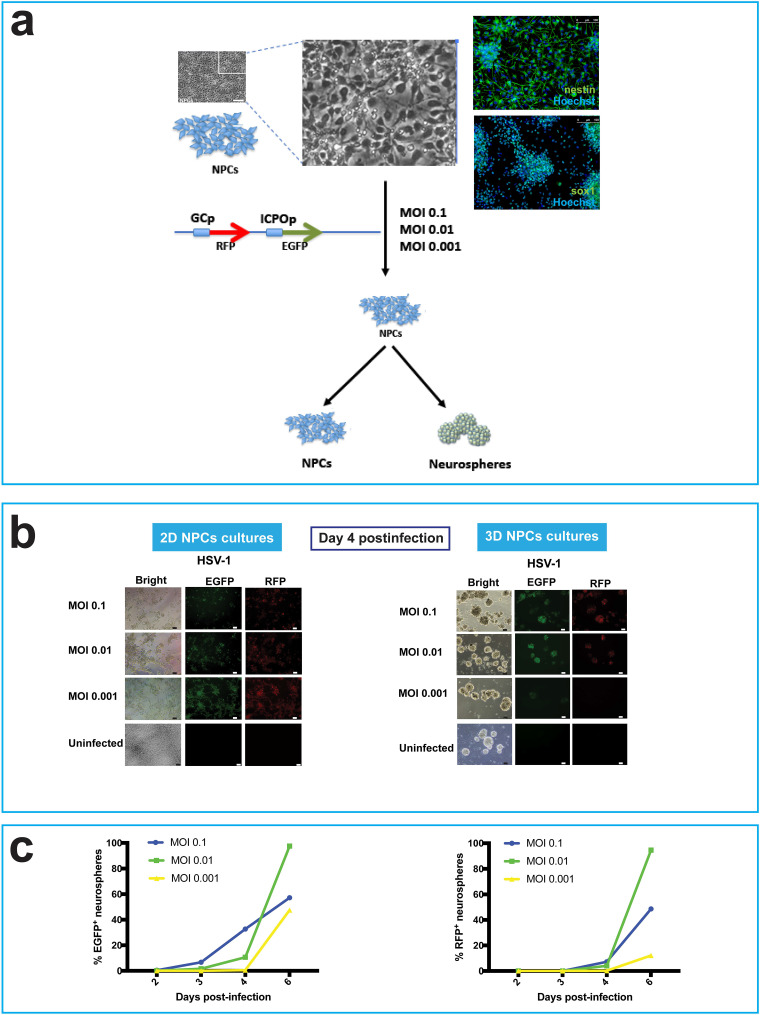
3D cultures of hiPSC-derived neuronal progenitor cells (NPCs) are less susceptible to HSV-1 than 2D cultures. (a) Scheme to investigate the behavior of HSV-1 in 2D and 3D cultures of NPCs. HSV-1 expresses EGFP and RFP reporter genes under the control of the ICP0 and gC promoters, respectively. The expression of the NPC markers nestin and SOX1, visualized by immunocytochemistry, is shown. (b) Comparison of cell morphology and virus gene expression of hiPSC-derived NPCs infected with HSV-1 at a range of MOIs from 0.1 to 0.001 and cultured as 2D monolayer cultures (left panel) or 3D cell aggregates (neurospheres; right panel). At day 4 postinfection (p.i.), all 2D cell cultures expressed EGFP and RFP in most of the cells infected at different MOIs. The expression of the fluorescent reporter genes was delayed in the 3D cultures (neurospheres) generated from infected monolayer 2D NPC cultures. At day 4 p.i., only a fraction of neurospheres derived from NPCs infected at different MOIs expressed the fluorescent reporter genes. Bars, 50 μm. (c) Graphs depicting the percentage of neurospheres expressing the EGFP and RFP fluorescent reporter genes at different time points (days 2 to 6).

Human NPCs were derived from hiPSCs as previously described (16) or using the StemDiff SMADi neural induction kit (STEMCELL Technologies). Monolayer cultures of NPCs were infected at a range of low multiplicities of infection (MOIs) (0.1 to 0.001) with a recombinant HSV-1 expressing the reporter genes for enhanced green fluorescent protein (EGFP) and red fluorescent protein (RFP) under the control of the HSV-1 promoters ICP0 and gC, respectively. The infected cells were then either cultured as 2D monolayers or dissociated and transferred to ultralow-attachment plates to allow the formation of 3D spherical cell aggregates, termed neurospheres (Fig. 1a).

At day 4 postinfection, most cells in 2D cultures expressed EGFP and RFP in cultures infected at MOIs ranging from 0.1 to 0.001 (Fig. 1b). Indeed, cells infected at MOIs of 0.1 and 0.01 detached from the bottom surface of the culture wells, which is characteristic of HSV-1 cytopathic effect in typical permissive epithelial cell cultures (Fig. 1b). Conversely, in 3D cultures (neurospheres) generated from infected monolayers of 2D NPC cultures, the expression of the fluorescent reporter genes was delayed (Fig. 1b and c). At day 4 postinfection, only a fraction of neurospheres derived from infected NPCs expressed the fluorescent reporter genes infected at different MOIs. We estimated the following fractions of fluorescent cells at each multiplicity: at an MOI of 0.1, 32.7% EGFP^+^ and 7.2% EGFP^+^ RFP^+^ neurospheres; at an MOI of 0.01, 10.6% EGFP^+^ and 4% EGFP^+^ RFP^+^ neurospheres; at an MOI of 0.001, 0.5% EGFP^+^ and 0.17% EGFP^+^ RFP^+^ neurospheres (Fig. 1c).

Loss of structural integrity of the fluorescent neurospheres was observed at an MOI of 0.1 but not at lower MOIs (Fig. 1b). Taken together, these results indicate that 3D spherical aggregates are less susceptible to a productive HSV-1 infection than 2D monolayer cultures.

### HSV-1 establishes persistent infection in NPCs at low MOIs.

**FIG 2:**
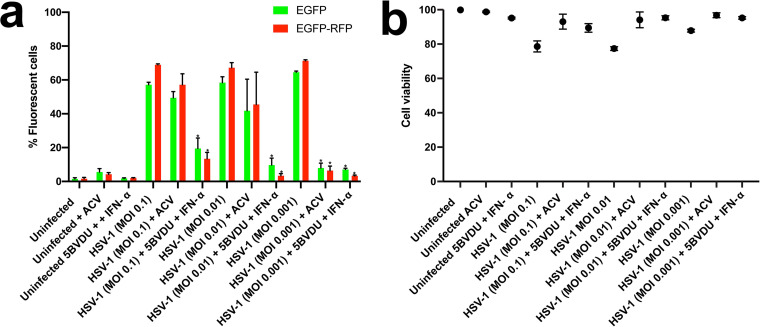
Analysis of uninfected and HSV-1-infected NPCs exposed to the antivirals acyclovir and 5BVdU–IFN-α. Antiviral activity (a) and cytotoxicity (b) of acyclovir (ACV) and 5BVdU–IFN-α in NPCs infected with HSV-1 expressing the EGFP and RFP reporter genes under the control of the viral promoter genes ICP0 and gC, respectively, at MOIs of 0.1 to 0.001 are shown. Student’s *t* tests were conducted between treated and untreated infected cultures and showed significant differences between infected cultures treated with 5BVdU–IFN-α and those not treated at MOIs of 0.1 to 0.001. Infected cultures treated with ACV showed a significant reduction in the percentage of infected cells only at an MOI of 0.001. *, *P* < 0.0001.

Culturing neuron cell types in the presence of antiviral drugs blocks lytic infections, enabling analysis of latent infections. We previously provided evidence of the ability of HSV-1 to establish latency in hiPSC-derived central nervous system (CNS) neurons using this approach (7, 17). In this study, we investigated whether the establishment of HSV-1 latency can be established in NPCs, which represent earlier stages of neuronal differentiation. Thus, we explored whether NPCs infected with HSV-1 and coincubated with acyclovir (ACV) at 50 μM or a cocktail consisting of (E)-5-(2-bromovinyl)-2′-deoxyuridine (5BVdU), a thymidine nucleoside analogue which acts as a competitive inhibitor of viral DNA polymerase (18) (30 μM), and alpha interferon (IFN-α; 125 U/ml) exhibit key landmarks of HSV-1 latency. We previously used this cocktail to induce latent states and block lytic replication in model 2D neuron systems (7, 17). Monolayer cultures of NPCs were infected at a range of low multiplicities of infection (0.1 to 0.001) with the HSV-1 construct expressing the EGFP and RFP reporter genes and cultured in the presence of the aforementioned antivirals. At day 7 postinfection (p.i.), flow cytometry analysis showed a significantly reduced percentage of infected NPCs (*P* < 0.05) in cultures exposed to the combination 5BVdU–IFN-α compared to untreated cells infected for 48 h, but the reduction of infected cells in ACV-treated cultures did not reach statistical significance at MOIs of 0.1 and 0.01 (Fig. 2a). Intriguingly, while a fraction of cells showed expression of HSV-1 proteins at higher MOIs, the vast majority of NPCs remained viable, as determined by a viability dye assay with 780. The cell viability of ACV-treated cultures infected at different MOIs was comparable to that of 5BVdU–IFN-α-treated NPCs infected at the same MOIs (Fig. 2b). These results indicate that while HSV-1 infects a substantial percentage of the NPCs and the immediate early and late promoters express their fluorescent reporters in a large number of NPCs, the majority of the cells remain viable, even in the absence of antivirals, as determined by using a viability assay.

**FIG 3:**
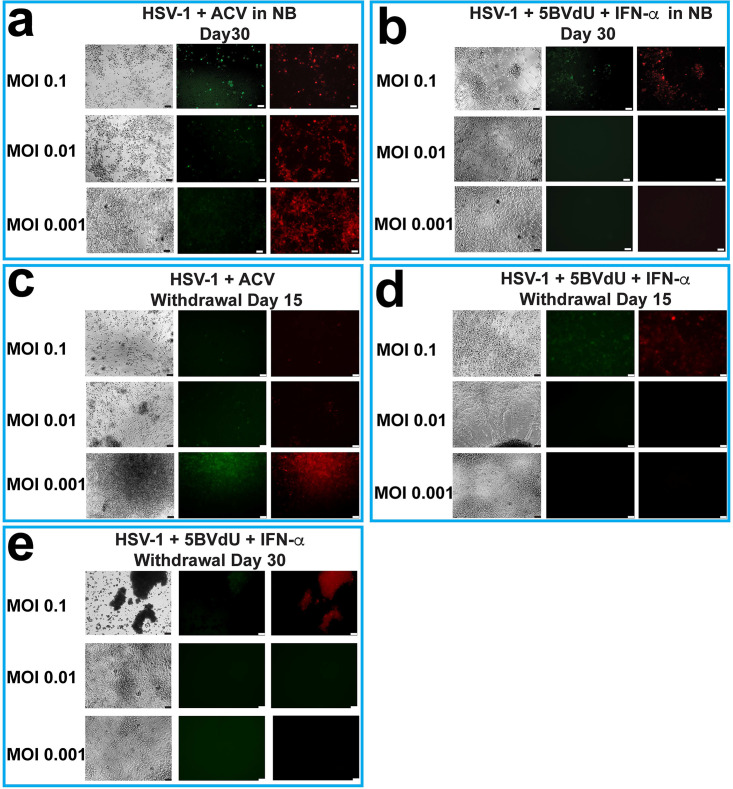
Acyclovir shows reduced antiviral efficacy in HSV-1-infected NPCs compared to 5BVdU–IFN-α. Microphotographs of hiPSC-derived NPCs, at MOIs of 0.1 to 0.001 in the presence of the antiviral acyclovir or 5BVdU–IFN-α. (a and b) The infected cells were cultured in neurobasal medium in the presence of antivirals for 30 days. (c to e) In a parallel experiment, NPCs infected at MOIs of 0.1 to 0.001 in the presence of the aforementioned antivirals were cultured in neuronal progenitor medium. At day 7 postinfection (p.i.), the antivirals were withdrawn and cells were cultured in neurobasal medium. The microphotographs depict the expression of fluorescent cells in infected cultures after withdrawal of the antivirals for 15 (c and d) and 30 (e) days. Bars 50 μm.

Next, we investigated the behavior of HSV-1 in NPCs exposed to ACV or to 5BVdU–IFN-α during neuronal differentiation. Monolayer cultures of NPCs were pretreated with the antiviral ACV or 5BVdU–IFN-α for 24 h, after which they were infected at a range of low multiplicities of infection (0.1 to 0.001) in the presence or absence of antivirals with the aforementioned viral construct. After 24 h, the infected cells were cultured in neurobasal medium supplemented with B27 and brain-derived neurotrophic factor (BDNF) to induce neuronal differentiation of the NPCs, along with maintained levels of the antivirals. The expression of the fluorescent reporter genes was then monitored by fluorescence microscopy. At day 30 postinfection, all the cells in the ACV-treated cultures were EGFP^+^ and RFP^+^ (Fig. 3a). Conversely, fluorescent cells were detected in a visibly lower proportion in 5BVdU–IFN-α-treated cultures infected at MOIs of 0.01 and 0.001 (Fig. 3b).

In a parallel experiment, NPCs were pretreated with ACV or 5BVdU–IFN-α for 24 h, infected with the HSV-1 recombinant expressing the EGFP and RFP reporter genes at MOIs of 0.1 to 0.001 as described above, and cultured in neural progenitor medium in the presence of antivirals for 7 days. Then, antivirals were withdrawn, and the NPCs were cultured in neurobasal medium to induce neuronal differentiation. Fifteen days after antiviral withdrawal, all the cultures infected at different MOIs and treated with ACV expressed the EGFP and RFP reporter genes (Fig. 3c); conversely, at this time point in 5BVdU–IFN-α-treated cells, the expression of the reporter genes was observed only in a fraction of cells infected at an MOI of 0.1 (Fig. 3d). At day 30 after antiviral withdrawal, EGFP^+^ and RFP^+^ cells were detectable in only a few single cells infected at the MOI of 0.01, and none were seen at the lower MOI (Fig. 3e).

On the basis of these results, the antiviral combination 5BVdU–IFN-α was used in subsequent experiments to prevent lytic infections from spreading in setting up studies of latency. We also investigated whether patterns of infection differed between 2D and 3D NPC cultures. The generation of infected 2D and 3D NPC cultures after infection at MOIs of 0.1 to 0.001 in the presence or absence of 5BVdU–IFN-α was therefore examined.

**FIG 4:**
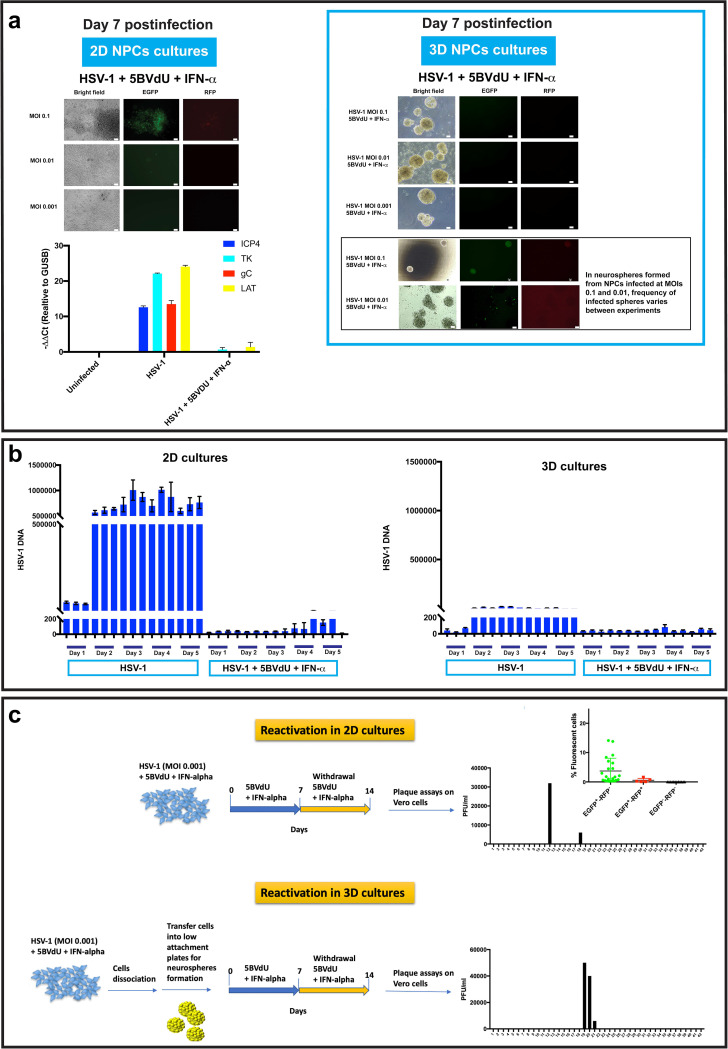
Persistent HSV-1 infection is established in NPCs. (a) (Top left) Comparison between infected 2D and 3D NPC cultures (neurospheres) exposed to the antivirals 5BVdU–IFN-α. At day 7 p.i., the antivirals 5BVdU–IFN-α efficiently inhibited HSV-1 replication at an MOI of 0.001 in 2D monolayer cultures. (Bottom left) RT-qPCR analysis shows that treatment of HSV-1-infected 2D NPC cultures with 5BVdU–IFN-α causes a significant downregulation of viral genes (*P < *0.0001 for all comparisons). (Top right) In 3D cultures exposed to antivirals, at day 7 postinfection, EGFP^+^ or EGFP^+^ RFP^+^ cells were not observed in cultures infected at an MOI of 0.001 and were observed at a variable frequency at MOIs of 0.1 and 0.01. Examples of neurospheres carrying EGFP^+^ and RFP^+^ cells are depicted in the black box. (b) Time course quantification of viral DNA in 2D and 3D cultures infected at an MOI of 0.001 in the presence or absence of antivirals from days 1 to 5. (c) Analysis of productive infection in 2D and 3D NPC cultures after withdrawal of the antivirals. NPCs were infected at an MO1 of 0.001 in the presence of antivirals and cultured as 2D or 3D cultures, as described above. After 7 days, the antivirals were withdrawn and cells were cultured for an additional 7 days in the NPC culture medium. The presence of infectious viral particles in the culture medium was investigated by plaque assay in Vero cells. The expression of the fluorescent reporter genes 7 days after withdrawal of the antivirals in 2D cultures was investigated by flow cytometry.

In 2D monolayer cultures, 5BVdU–IFN-α efficiently inhibited HSV-1 replication, particularly at lower MOIs. At an MOI of 0.1, both red- and green-fluorescing cultures were seen, but at MOIs of 0.01 and 0.001, EGFP^+^ and EGFP^+^ RFP^+^ cells were largely undetectable (Fig. 4a, top left). Analyses of RNA from such cultures by reverse transcription-quantitative PCR (RT-qPCR) showed a vastly significant reduction (*P < *0.0001) of the expression of the viral genes for ICP4, thymidine kinase (TK), gC, and the latency-associated transcript (LAT) at 7 days p.i. in 2D cultures infected in the presence of 5BVdU–IFN-α, compared to cells infected for 48 h in the absence of antivirals (Fig. 4a, bottom left).

When the 3D cultures were examined, expression of EGFP and RFP was not detected at an MOI of 0.001 at day 7 postinfection in the presence of the antivirals, although both fluorescent genes were detected in individual cells with variable frequency among different experiments in cultures infected at MOIs of 0.1 and 0.01 (Fig. 4a, top right).

In the experiments just detailed, there was an apparent complete inhibition of HSV-1 (based on fluorescent gene expression) in both 2D and 3D cultures infected at an MOI of 0.001 in the presence of 5BVdU–IFN-α. We chose this MOI to perform a time course quantification of viral DNA levels in 2D and 3D cultures in the presence or absence of antivirals. Cell lysates were prepared from uninfected and infected 2D and 3D NPC cultures from days 1 to 5 and used for quantitative DNA PCR (qPCR). In 2D cultures in the absence of antivirals, viral DNA levels rapidly increased by day 2 and remained high. A very modest increase of viral DNA was observed starting from day 4 in the presence of antivirals (Fig. 4b). In the 3D cultures, the overall antiviral activity of 5BVdU–IFN-α was more pronounced, and virtually no increase in viral DNA was seen (Fig. 4b, right). Intriguingly, even in the absence of antivirals, there was a much smaller viral DNA increase over the 5-day course. This is consistent with a preference for viruses to enter the latent state.

Next, we investigated whether the withdrawal of antivirals was followed by active viral replication in the cell culture models, representing spontaneous reactivation events. NPCs were infected at an MO1 of 0.001 in the presence of antivirals and cultured as 2D or 3D cultures as described above for 7 days, and then the antivirals were withdrawn. Cells were cultured for an additional 7 days in the NPC culture medium. The presence of infectious viral particles in the culture medium was investigated by plaque assay in Vero cells. Productive infection was observed in only 2 of 42 2D cultures and in 3 of 42 3D cultures (Fig. 4c). However, a flow cytometry analysis of the 2D cultures showed that various percentages of EGFP^+^ cells developed (0.79% to 21%), occurring in 25 of 42 2D NPC cultures 7 days after withdrawal of the antivirals. EGFP^+^ RFP^+^ cells were detected in 5 of 42 cultures (0.11% to 1.63% [Fig. 4c]), indicating that some type of incomplete reactivation occurred in ∼50% of the infected cultures.

We then evaluated reactivation stimuli that have been documented to induce reactivation in other models. The exposure of both 2D and 3D cultures to the phosphatidylinositol 3-kinase inhibitor (PI3Ki) LY294002 (20 μM) for 7 days to induce viral reactivation did not lead to any increase in the fraction of culture wells exhibiting renewed expression of the fluorescent reporters (data not shown). Together, these results indicate that in NPCs infected at an MOI as low as 0.001, HSV-1 can establish a silenced state that is relatively refractory to reactivation stimuli that are known to induce reactivation in sensory neuron models.

### HSV-1 affects NPC migration.

**FIG 5:**
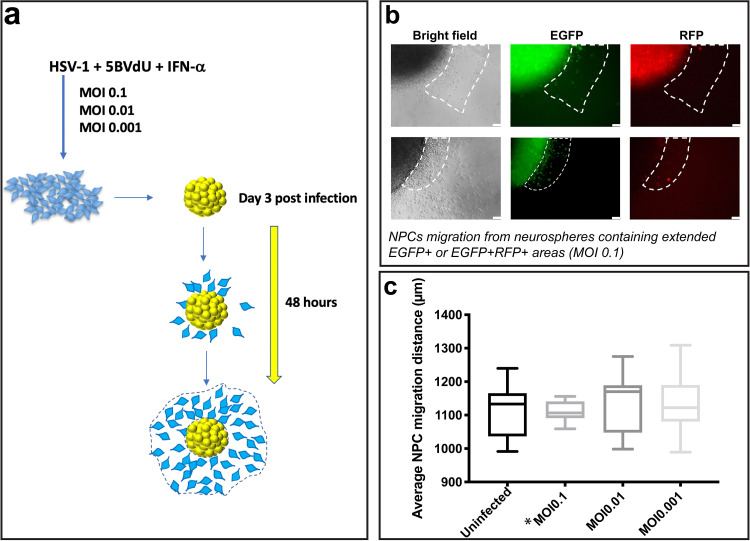
HSV-1 affects NPC migration in a dose-dependent fashion in the presence of antivirals. (a) NPCs were infected at MOIs of 0.1 to 0.001 in the presence of 5BVdU–IFN-α and cultured as 3D cultures (neurospheres). Seventy-two hours later, neurospheres generated from uninfected and infected NPCs were transferred to Matrigel-coated 12-well plates to allow the NPCs to migrate from the neurosphere. (b and c) NPC migration was analyzed after 48 h. (b) A fraction (approximately 45%) of neurospheres derived from NPCs infected at an MOI of 0.1 were EGFP^+^ or EGFP^+^ RFP^+^, as estimated by direct count of fluorescent neurospheres using fluorescence microscopy. Representative microphotographs depicting NPC migration from these fluorescent neurospheres are shown. Dotted lines demarcate the areas containing the migrating EGFP^+^ and EGFP^+^ RFP^+^ cells. (c) The radial migration of NPCs from neurospheres containing a low proportion of EGFP**^+^** RFP^−^ cells in cultures infected at an MOI of 0.1 and neurospheres from uninfected cultures or cultures infected at MOIs of 0.01 to 0.001 was assessed. Error bars show standard deviations. A one-way analysis of variance (ANOVA) followed by Bonferroni’s *post hoc* test showed no statistically significant difference in cell migration between any two conditions. Asterisk indicates neurospheres containing a low proportion of EGFP**^+^** RFP^−^ cells in cultures infected at an MOI of 0.1.

A fundamental aspect of adult neurogenesis is the ability of NPCs to migrate great distances through the parenchyma to reach damaged areas of the brain (19). Neurotropic viruses, such as HIV, human cytomegalovirus (HCMV), and Zika virus (20–23), are known to alter migration behavior of NPCs, but the effect of HSV-1 on NPC migration is unknown. To investigate the effect of HSV-1 on NPC migration and whether HSV-1-induced alteration of NPC migration can be prevented by antiviral treatment, NPCs were infected at MOIs of 0.1 to 0.001 in the presence of 5BVdU–IFN-α. Cells from different conditions were dissociated and transferred to ultralow-attachment plates for the formation of neurospheres (see Materials and Methods). Cells under all conditions were cultured in the presence of antivirals. Seventy-two hours later, neurospheres generated from uninfected and infected NPCs were transferred individually into Matrigel-coated 96-well plates to allow the NPCs to migrate. After 48 h, the greatest cellular migration of NPCs from each neurosphere was assessed (Fig. 5a), as described in Materials and Methods.

In the cultures infected at an MOI of 0.1, we observed either neurospheres containing high proportions of EGFP**^+^** RFP**^+^** cells (45% of spheres) or neurospheres containing a very low proportion of detectable EGFP**^+^** RFP**^+^** cells. A clear effect of lytic infection on NPC migration was observed. In fact, from the former group, cells expressing both EGFP and RFP could migrate a short distance (less than 250 μm, while the average distance migrated by EGFP^−^ RFP^−^ NPCs was 1.1 mm) (Fig. 5b). From the latter group, all the migrating cells were EGFP^−^ RFP^−^. Furthermore, no significant differences in NPC migration (*P = *0.89) were observed between the latter group at an MOI of 0.1 (neurospheres containing a very low proportion of EGFP^+^ RFP^−^ cells) and neurospheres generated from NPCs infected at MOIs of 0.01 to 0.001 or uninfected NPCs (Fig. 5c).

Collectively, these results suggest that (i) HSV-1 lytic infection impairs NPC migration and (ii) that the protective effect of the antivirals 5BVdU–IFN-α is viral dose dependent.

## DISCUSSION

Herpes simplex virus 1 (HSV-1) can establish latent infections in peripheral nerve ganglia and the central nervous system (CNS) of experimentally infected mice. A characteristic of latency is the ability to recover infectious virus from tissues where latency is established. Infectious virus can be successfully recovered from peripheral ganglia but not from the CNS of latently infected mice following explant cultures (24), although viral DNA can be detected in different CNS regions. The inability to detect viral reactivation from the CNS has been interpreted for decades as an indication that CNS is not a source of HSV-1 latency. Alternative explanations were provided by Deatly et al., who suggested that the difference in reactivation between peripheral nervous system and CNS tissues may reflect differences in the host nervous system tissues, density of latently infected cells, and cellular factors (25). Using a modified *ex vivo* tissue explant reactivation assay (i.e., the dissociation method instead of the classical mincing method), Chen et al. achieved efficient recovery from the brain stem, olfactory bulb, and frontal cortex but not from the hippocampus, demonstrating that CNS can be a site of latency for HSV-1 (26). Recent studies have provided evidence that HSV-1 can establish latency in CNS neurons (17, 27–29). However, it is uncertain whether neurons constitute the only reservoir of latent HSV-1 in the CNS. The exclusivity of the neurons as the principal sites for HSV-1 latency implies that the ability of the virus to establish latency is gained during the neuronal differentiation of NPCs or neuronal maturation. Considering the tropism of HSV-1 for the neurogenic niches in the CNS, in this study we sought to investigate whether NPCs can harbor latent viral genomes.

We and others have reported on the susceptibility of NPCs to HSV-1 infection (7, 8, 14). In this study, we employed hiPSCs to model aspects of the interaction of HSV-1 with NPCs when cultured as 2D monolayers or as 3D spherical aggregates (neurospheres). The necessity of comparing 2D versus 3D cultures stemmed from the increasing evidence of the superiority of the latter to model host-pathogen interaction. Our results show a reduced susceptibility of 3D NPC cultures to acute HSV-1 infection (Fig. 1b).

We compared the antiviral activities of ACV and of 5BVdU–IFN-α in infected NPCs. 5BVdU (also known as brivudin) is the most efficacious antiviral against varicella-zoster virus (VZV) infections and has also been used to treat HSV-1 infections (18). It is currently available in several European countries but not in the United States. We found that the combination of 5BVdU–IFN-α is superior to ACV, as this combination effectively inhibited HSV-1 replication and gene expression in hiPSC-derived NPCs (Fig. 3a). The withdrawal of 5BVdU–IFN-α from infected differentiating NPCs did not lead to viral reactivation after 30 days in cultures infected at an MOI of 0.001 (Fig. 3e). Conversely, all the ACV-treated NPCs were EGFP^+^ and RFP^+^ after the antiviral withdrawal for 15 days (Fig. 3c). At MOIs of 0.1 and 0.01, the inhibitory effect of 5BVdU–IFN-α was more pronounced in 3D cultures (Fig. 4a). These results highlight the importance of 3D cultures when host-pathogen interactions are being modeled.

The efficient anti-HSV-1 activity of 5BVdU–IFN-α in NPC cultures infected at an MOI of 0.001 raised the possibility that HSV-1 latency can be established in NPCs. Evidence of viral lytic genes and LAT (a key landmark of HSV-1 latency) expression in NPCs of the SVZ has been observed in latently infected mice at day 30 postinfection (14), but it is not clear whether the expression of these viral genes underlies latent or chronic infection.

Plaque assays in Vero cells showed that in infected 2D and 3D NPCs (MOI, 0.001) cultured in the presence of 5BVdU–IFN-α for 7 days, the withdrawal of the antivirals led to viral reactivation in only 4.7% and 7.1% of the cultures, respectively. Comparative gene expression analysis indicated a drastic reduction of the expression of the viral genes for ICP4, thymidine kinase (TK), and gC in infected NPCs exposed to 5BVdU–IFN-α for 7 days compared to acutely infected cells (Fig. 4a). In fact, high levels of the LAT transcripts were detected at 48 h p.i. At 7 days p.i., LAT expression was decreased compared to its expression at the 48-h time point. This result is consistent with the lower levels of LAT expression that are observed in CNS neurons *in vitro* (17) and *in vivo* (30) than in neurons of the sensory ganglia. Also, these results are in line with those reported by Menendez et al. (14) showing LAT expression in the ependyma, which harbors NPCs, that was significantly lower than that in trigeminal ganglia in latently infected mice at 30 days postinfection.

Flow cytometry analysis showed EGFP^+^ RFP^−^ cells in approximately 48% of cultures, indicating abortive reactivation, while a small fraction of EGFP^+^ RFP^+^ cells (0.11 to 1.63%) was detected in approximately 12% of the cultures (Fig. 4c). Together, these results suggest that HSV-1 can establish largely silenced persisting DNA in NPCs infected at an MOI as low as 0.001. We hypothesize that (i) a substantial fraction of viral genomes in infected NPCs exposed to 5BVdU–IFN-α undergo a form of silencing that is relatively refractory to reactivation and (ii) this viral silencing mechanism can be a variant of the classical HSV-1 latency where specific epigenetic modifications of the viral genomes and/or host-specific mechanism/s do not favor the heterochromatin-euchromatin transition. The veracity of this hypothesis would imply that the ability of HSV-1 to efficiently reactivate from latency is gained during the neuronal differentiation of NPCs or neuronal maturation.

It is important to note that in hiPSC-derived neurons, latency can be established at an MOI of 0.3 (7, 17). This observation provides further evidence for the higher susceptibility of NPCs than of neurons to HSV-1. These data are particularly relevant in light of recent data that associate HSV-1 with Alzheimer’s disease (31) and a recent report that showed accumulation of beta amyloid in neural stem cells harboring HSV-1 lytic infection (32).

The results summarized in Fig. 5 suggest that, even in the presence of 5BVdU–IFN-α, HSV-1 impairs NPC migration (which represents a critical step in neurogenesis) in a viral-dose-dependent fashion. This evidence poses the need to develop novel antiherpetic drugs to protect neurogenesis from the HSV-1 inhibitory effect.

### Conclusions.

We employed hiPSCs to model aspects of the HSV-1–NPC interaction in the presence and absence of antiviral drugs. We found that HSV-1 can establish persistent infection in NPCs, suggesting that neurogenic niches in the brain may constitute additional sites where viral reactivation may occur. Furthermore, we observed reduced susceptibility of 3D NPC cultures to HSV-1 compared to 2D cultures, highlighting the potential value of 3D cultures for modeling host-pathogen interactions. Our results suggest the relative superiority of 5BVdU–IFN-α to ACV to block viral replication in NPCs. Our data also indicate that the silenced viral genomes in NPCs may not respond to the PI3 signaling inhibition triggers that drive reactivation in sensory neurons. Indeed, the triggers of reactivation in this cell type will be important to resolve and define and they may be quite different from that in the sensory neuron phenotype. These data are relevant for recent observations relating HSV-1 infection to postencephalitic cognitive dysfunction and even Alzheimer’s disease (AD).

## MATERIALS AND METHODS

### Cell lines.

Vero cells (CCL-81; ATCC) for plaque assay were maintained in Eagle’s minimum essential medium (EMEM) supplemented with 10% fetal bovine serum (FBS; HyClone) and 5% antibiotic/antimycotic (HyClone).

The hiPSC line 73-56010-02 was employed in this study. hiPSC line 73-56010-02 was generated from fibroblasts derived from skin biopsy samples that were collected from a healthy individual via 4-mm full-thickness punch biopsies under local anesthesia. The hiPSCs were established at the National Institute of Mental Health (NIMH) Center for Collaborative Studies of Mental Disorders-funded Rutgers University Cell and DNA Repository (http://www.rucdr.org/mental-health) (RUCDR).

All cells were cultured under standard conditions (37°C, 5% CO, and 100% humidity).

### Viral infections.

The following HSV-1 strains were employed in this study: KOS (VR-1493; ATCC) and a KOS-based recombinant construct incorporating the genes for enhanced green fluorescent protein (EGFP) and monomeric red fluorescent protein (RFP) as reporter genes whose expression is driven by the viral promoters ICP0 and glycoprotein C, respectively. This KOS-based recombinant HSV-1 construct was developed on the KOS strain originally obtained from a master stock originally obtained from P. Schaffer in a manner similar to that detailed previously (33). The KOS strain used has been sequenced (GenBank accession no. JQ780693.1). Gene cassettes were developed to recombine into the nonessential gC locus found in UL44, which does not influence the pathogenesis of HSV-1 KOS in the mouse corneal scarification infection model (33). The plasmid vector pgCp-EGFP was previously detailed and contains the gC promoter driving expression of EGFP inserted in frame with the ATG of UL44, followed by a translational stop, a poly(A) signal, and part of the gC open reading frame (ORF) in the vector pUC19 (34). The ICP0 promoter was generated by PCR and contained 600 bp of sequence immediately upstream of the ICP0 ATG, using the primers ICP0p HSV-1 promoter (F) GGATCCGGATCCGGGTCGTATGCGGCTGGAG and ICP0p HSV-1 promoter (R) AAGCTTAAGCTTACTTGCAAGAGGCCTTGTTC. The primers contained additional sequences to add a flanking unique HindIII site distally and a unique BamHI site at the proximal end of the promoter. These were cloned into the vector gCp-EGFP cut with BamHI and HindIII. Then, DNA containing the monomeric red fluorescent protein (mRFP) sequence with its bovine growth hormone (BGH) poly(A) signal was generated from the plasmid pgC-RFP or pgB-EGFP, detailed previously (35), and cloned into unique NheI and HindIII sites at the distal end of the ICP0 promoter, resulting in the placement of mRFP-poly(A) downstream of the gC promoter and upstream of the ICP0 EGFP gene. In summary, the construct contained, at the gC locus, the promoter of gC driving the expression of the mRFP, followed by a BGH poly(A) signal, and then the ICP0 promoter sequence driving EGFP. The flanking gC promoter and following open reading frame provide sequences for homologous recombination into the gC locus. Recombinant viruses were derived by cotransfection of linearized plasmids (using SspI or EcoRI) with HSV-1 KOS S1L infectious DNA prepared as detailed previously (33, 36). Virus plaques were selected based on the gain of red fluorescence and plaque purified to homogeneity. Each virus was grown and purified from Vero cells (36). Final recombinant sequences were verified by sequencing insertional junctions, and recombinant promoters and EGFP location were verified by Southern blotting.

### (i) Generation of neurospheres.

Human NPCs were derived from hiPSCs as previously described (16). Critical experiments were repeated using NPCs derived from hiPSCs using the StemDiff SMADi neural induction kit (STEMCELL Technologies). NPCs were seeded in Matrigel-coated 12-well plates at a density of 3 × 10^5^ cells/well and cultured in StemDiff neural progenitor medium until cells were approximately 80 to 85% confluent. NPCs were infected with the aforementioned KOS-based HSV-1 construct (at MOIs of 0.1 to 0.001) in the presence or absence of acyclovir (ACV, 50 μM) or of (E)-5-(2-bromovinyl)-2′-deoxyuridine (5BVdU; 30 μM) plus alpha interferon (IFN-α; 125 U/ml) and cultured in STEMdiff neural progenitor medium. NPCs that were infected in the presence of ACV were pretreated with the antiviral for 24 h. Infections were performed in triplicate. One hour after the infection, inocula were removed and cells were washed. Uninfected and infected NPCs were dissociated using Accutase and seeded into low-attachment 12-well plates at a density of approximately 2 × 10^4^ cells/well to allow the formation of neurospheres. The expression of the fluorescent reporter genes in the neurospheres was analyzed with a fluorescence microscope on a daily basis.

### (ii) NPC migration assays.

To assess NPC migration, NPCs were infected at the MOIs reported above in the presence or absence of 5BVdU–IFN-α. At 1 h postinfection, the inocula were removed, cells were washed, and uninfected and infected NPCs were dissociated and transferred at a density of 2 × 10^4^ cells/well into low-attachment round (U)-bottom 96-well plates for the formation of neurospheres (one in each well) with comparable sizes. The uniformity of size of the neurospheres is desirable for comparing cell migrations under different conditions. Seventy-two hours later, neurospheres generated from uninfected and infected NPCs were transferred to Matrigel-coated 12-well plates. After 48 h, the radial migration of NPCs from each neurosphere was assessed by calculating the radius of the surface generated after manual tracing of the edge of the furthest-migrating NPCs using NIH ImageJ (37). A minimum of 10 neurospheres per condition were analyzed.

### Flow cytometry.

Flow cytometry with BD LSRFortessa was employed for quantitative analysis of fluorescent cells and cell viability. The cell viability was assayed using the viability dye 780 (BioGems, Ltd.).

### qPCR.

Viral DNA was quantified by real-time PCR using primers specific for the EGFP reporter gene from the genetically engineered HSV-1 construct. The infected and uninfected NPCs in 24-well plates were lysed with 150 μl of QuickExtract DNA buffer (Lucigen). For each reaction, 2 μl of cell lysate was added to 8 μl master mix composed of TaqMan master mix (catalog no. 4370048; Thermo Fisher), 18 μM forward primer (5′-CCACATGAAGCAGCACGACTT-3′), 18 μM reverse primer (5′-GGTGCGCTCCTGGACGTA-3′), and 18 μM internal oligonucleotide (5′-FAM [6-carboxyfluorescein]-TTCAAGTCCGCCATGCCCGAA-TAMRA [6-carboxytetramethylrhodamine]-3′). The following qPCR conditions were used: 95°C for 3 min followed by 45 cycles of 95°C for 15 s and 55°C for 30 s, finishing with 72°C for 30 s.

A plasmid containing the EGFP target sequence was used to create a standard curve of the cycle threshold (*C_T_*) values from serial dilutions of the plasmid (8 × 10^7^ to 1 copy/well). The *C_T_* values of the unknown samples were plotted on the standard curve, and copy numbers were determined and normalized to those of human GAPDH. Each sample and standard was analyzed in triplicate.

### RT-qPCR.

RNA from 2D NPC cultures was extracted using an RNeasy Plus kit (catalog no. 74134; Qiagen). Reverse transcription was performed with random primer mix using a ProtoScript II first-strand cDNA synthesis kit (catalog no. E6560S; New England BioLabs, Inc.) following the manufacturer’s instructions.

The qPCR was performed using Applied Biosystems TaqMan gene expression master mix (catalog no. 4369016; Thermo Fisher Scientific). The following primers were used: LAT intron F (5′-CGCCCCAGAGGCTAAGG-3′), LAT intron R (5′-GGGCTGGTGTGCTGTAACA-3′), LAT intron probe (5′-CCACGCCACTCGCG-3′), ICP4 F (5′-CACGGGCCGCTTCAC-3′), ICP4 R (5′-GCGATAGCGCGCGTAGA-3′), ICP4 probe (5′-CCGACGCGACCTCC-3′), TK F (5′-CACGCTACTGCGGGTTTATATAGAC-3′), TK R (5′-GGCTCGGGTACGTAGACGATAT-3′), TK probe (5′-CACCACGCAACTGC-3′), gC F (5′-CCTCCACGCCCAAAAGC-3′), gC R (5′-GGTGGTGTTGTTCTTGGGTTTG-3′), and gC probe (5′-CCCCACGTCCACCCC-3′).
