# Correction for Zheng et al., “Patterns of Herpes Simplex Virus 1 Infection in Neural Progenitor Cells”

**DOI:** 10.1128/JVI.02189-20

**Published:** 2021-01-28

**Authors:** Wenxiao Zheng, Alissa M. Klammer, Jennifer N. Naciri, Jason Yeung, Matthew Demers, Jadranka Milosevic, Paul R. Kinchington, David C. Bloom, Vishwajit L. Nimgaonkar, Leonardo D’Aiuto

**Affiliations:** a Department of Psychiatry, Western Psychiatric Institute and Clinic, University of Pittsburgh School of Medicine, Pittsburgh, Pennsylvania, USA; b Third Xiangya Hospital, Xiangya School of Medicine, Central South University, Changsha, China; c University of Pittsburgh, Department of Infectious Diseases and Microbiology, Pitt Graduate School of Public Health, Pittsburgh, Pennsylvania, USA; d Captis Diagnostics, Pittsburgh, Pennsylvania, USA; e Carnegie Mellon University, Pittsburgh, Pennsylvania, USA; f Department of Ophthalmology, University of Pittsburgh School of Medicine, Pittsburgh, Pennsylvania, USA; g Department of Molecular Microbiology and Genetics, University of Pittsburgh, Pittsburgh, Pennsylvania, USA; h Department of Molecular Genetics & Microbiology, University of Florida College of Medicine, Gainesville, Florida, USA

## AUTHOR CORRECTION

Volume 94, no. 16, e00994-20, 2020, https://doi.org/10.1128/JVI.00994-20. Page 12: The following paragraph should be added to Acknowledgments. “We acknowledge the following funding sources: grant 1R21NS096405-01A1 from the National Institute of Neurological Disorders and Stroke (NINDS) (Leonardo D’Aiuto), grant 5R01AI122640-05 from the National Institute of Allergy and Infectious Diseases (NIAID) (Paul R. Kinchington), grant 2P30EY008098-31 from the National Eye Institute (NEI) (Paul R. Kinchington), grants 5T32AI007110-35 and 5R01AI048633-16 from the National Institute of Allergy and Infectious Diseases (NIAID) (David C. Bloom), grant 5R01MH063480-14 from the National Institute of Mental Health (NIMH) (Vishwajit L. Nimgaonkar), and grant 07R-1712 from the Stanley Medical Research Institute (SMRI) (Vishwajit L. Nimgaonkar).”

